# An Evidence-Based HIV Risk–Reduction Intervention for Young African American Women in the US South Using mHealth: Adaptation and Development Study

**DOI:** 10.2196/34041

**Published:** 2022-05-09

**Authors:** Rebecca L Watkins, Felicia A Browne, Paul N Kizakevich, Brittni N Howard, Leslie B Turner, Randall Eckhoff, Wendee M Wechsberg

**Affiliations:** 1 RTI International Research Triangle Park, NC United States; 2 Gillings School of Global Public Health The University of North Carolina Chapel Hill, NC United States; 3 Department of Psychology North Carolina State University Raleigh, NC United States; 4 Department of Psychiatry and Behavioral Sciences Duke University School of Medicine Durham, NC United States

**Keywords:** substance use, prevention, e-learning, adaptation, mobile apps, health risk behaviors, self-directed learning, HIV, women, young women, violence, mHealth app, gamification, mobile phone

## Abstract

**Background:**

Young African American women have higher rates of sexually transmitted infections, including HIV, than those of young women of other racial and ethnic groups. Gender-, culture-, and age-specific interventions are needed to end the HIV epidemic. The Women’s CoOp (WC) is an HIV risk–reduction intervention that is proven to be efficacious in various face-to-face formats.

**Objective:**

This study aims to adapt the delivery method of an evidence-based intervention, the WC, from an in-person format to a self-guided mobile health (mHealth) format while ensuring that core elements are maintained for intervention comparability and fidelity.

**Methods:**

Several adaptation phases were conducted by using the Personal Health Informatics and Intervention Toolkit (PHIT) as a guiding point to create the mobile app version of the WC. Throughout 5 phases, we established the implementation groundwork for the app; conducted formative research activities to test the initial draft of the app and obtain feedback; applied the PHIT toolkit programming structure to produce the mHealth version of the WC intervention; conducted usability testing and pretesting with interested parties, followed by in-house testing by WC interventionists and PHIT developers; and deployed the app to tablets and distributed it to study participants. The app underwent regular maintenance updates during the study.

**Results:**

The team converted the seven elements of the WC as accurately as possible for comparability to determine efficacy in a mobile app format while changing little about the basic delivery methods. For instance, *cue card* presentations of the materials delivered by the intervention staff were presented within the app but with voice-over narration and in a self-guided format rather than being led by a staff member. Other aspects of the intervention did not lend themselves to such straightforward adaptation, such as hands-on condom proficiency practice and one-on-one goal-setting activities. In these cases, the subject matter experts and app developers worked together to find comparable analogs to be used within the app. Once developed, tested, and finalized, the mHealth WC app was deployed into local health departments as part of a randomized trial.

**Conclusions:**

This systematic adaptation process created an accurate mHealth equivalent of an existing, in-person behavioral health intervention. Although participants’ reception of the app during the formative developmental phase was overall positive, maintaining fidelity to the in-person delivery compromised the natural capabilities of a mobile app, such as further gamification, different types of interactivity, and integrated notifications and messaging, which could be helpful for participants’ adherence to the intervention schedule. Given the development and implementation of the app, the next step is to examine the impact of the app and its efficacy in HIV and substance use risk-reduction.

## Introduction

### Background

African American women have experienced an unparalleled burden of HIV since the start of the epidemic and currently account for 55% of new HIV cases among women in the United States, despite representing 13% of women in the US population [1]. The rate of new HIV diagnoses among African American women is 13 times higher than that of White women and nearly 4 times higher than that of Hispanic or Latina women [2]. The disparity is even greater among African American female adolescents and young adults, particularly in the US South. In 2019, adolescents and young adults aged 13 to 24 years represented 21% of 36,398 individuals newly diagnosed with HIV in the United States [3]. In addition, approximately 51% of the new cases occurred in the US South, with African Americans representing 52% of these cases in the region [3].

Sexually transmitted infections (STIs) further place women at an increased risk for HIV, with African American women experiencing similar disparities in STI rates. Adolescents and young adults aged 15 to 24 years represent approximately half of the estimated 19 million new STI cases that occur each year in the United States, with young African American women experiencing approximately 4.5 times the rate of chlamydia, 5.2 times the rate of syphilis, and 9.3 times the rate of gonorrhea compared with young White women in the same age group [4]. These disparities in STI rates are further reflected in the US South, including North Carolina. Previous research has shown that social determinants, such as poverty, food insecurity, housing instability, lack of childcare, lack of transportation, experienced stigma, substance use, violence from intimate partners, and other socioeconomic constraints and structural barriers are associated with STI risk and acquisition [5].

Despite these alarming rates, few cultural-, age-, and gender-specific strategies exist to reach young African American women, which promote positive health behavior change by addressing social determinants and providing HIV risk–reduction information and skill-building activities. Consequently, technologically innovative methods of reducing STI and HIV burden based on adapted evidence-based HIV prevention strategies need to be tested with young African American women.

An estimated 97% of the US population owns a mobile phone, with approximately 83% of African Americans reporting smartphone ownership and 99% reporting ownership of any type of mobile phone [6]. Notably, African Americans have the highest smartphone ownership and use compared with any other racial or ethnic group in the United States [7]. Among African Americans, an estimated 85% of individuals text an average of 70 texts per day, with higher rates among female adolescents and young adults [6]. In addition, current research indicates that individuals with lower levels of income and education text more often than those at the higher end of the income and education scale [8-10].

The rapidly expanding accessibility of mobile technology, including smartphones and internet-based laptops or tablets, offers a unique opportunity for affordable linkage to health care and other valuable health information for hard-to-reach populations [11-13]. However, despite the large amount of time that young African American women engage with their smartphones, and given that racial and ethnic minority groups are using mobile phones to access health information more frequently than nonminority individuals, few targeted mobile health (mHealth) initiatives exist to reach young African American women, which are intended to increase their positive health outcomes, especially in HIV prevention [14].

Several behavioral health interventions have been developed and implemented for African American women that aim to reduce HIV, substance use, gender-based violence, and other related risks [15-20]. One such intervention developed specifically for African American women is the Women’s CoOp (WC), a *best-evidence*, empowerment-based, in-person, HIV risk–reduction behavioral intervention [21,22]. The WC is one of a few best-evidence interventions for African American women in the US South who use substances—a key population at risk for HIV. Originally, a 4-session cue card intervention comprising 2 individual and 2 group sessions focused on achieving personal power and agency to reduce sexual risk for HIV and STIs through hands-on activities such as condom demonstrations, sexual negotiation, and partner communication role-play and by learning about their bodies and reproductive health. This intervention showed statistically significant reductions in sexual risk and substance use [22] and has been adapted to a variety of contexts and settings in the United States, South Africa, Russia, and the Republic of Georgia [23]. The WC has been delivered by both trained clinic staff and project staff interventionists.

### Aim

As part of a crossover randomized trial, this study sought to test the efficacy of two delivery methods—face-to-face compared with mHealth—of an adapted version of the WC. This adaptation has reached female African American adolescents in the US South using the same information and methods as the original WC but adjusted to the context of a younger age group [24]. We aimed to develop a comparable mobile app version (mobile WC [mWC]) of the in-person WC program. The ability to tailor behavioral interventions to the culture, age, and gender of the intended population makes mHealth an innovative and valuable tool for reaching young African American women for HIV prevention.

This paper describes the adaptation process, activities, and rationalizations behind decisions made by behavioral health researchers and app programmers involved in the mHealth app development, the challenges encountered in the course of implementation, and insights gained about the process of converting in-person programs to mobile formats [25].

## Methods

### Adaptation Process and Agile Development

Although the adaptation process was iterative rather than linear, the conducted activities encompassed multiple phases, from the initial selection of the mHealth platform to the app’s maintenance.

#### Phase 1: Selection of the mHealth Platform

When deciding on a technological platform to adapt the intervention to an mHealth format, the behavioral health team, led by the developer of the WC, held 2 specific goals paramountly. First, it was important that the resultant app be accessible to and usable by all participants equally. Second, the participant information needed to be thoroughly secured. These 2 guiding intentions informed the ultimate decision to use the Personal Health Informatics and Intervention Toolkit (PHIT) platform to produce the app.

The PHIT platform is a software development framework that allows for rapid production of cross-platform, research-oriented custom mHealth apps [26]. This framework is most often used to build personalized health intervention apps using the subjective and objective measurement, assessment, and plan methodology [27]. The cross-platform aspect means that a single codebase can produce both a native Android app and a native iOS (Apple) app. Consequently, no participant would be turned away for having access to one type of device or another.

However, participant access to a reliable Wi-Fi internet connection, singular nonshared ownership of a private smartphone, and consistency of app appearance and performance across all devices were concerns shared by the team. Ultimately, these concerns led to the decision of providing participants with project-assigned devices preloaded with the app rather than installing the app on the participants’ own devices, ensuring more consistency and participant confidentiality throughout the study. By providing a device for use during the study rather than having participants use their own devices, we were able to rely on using one type of device and operating system to ensure each participant’s experience was the same from an equipment standpoint.

All research data collected and generated via the use of a PHIT app are stored locally within the app space in an encrypted SQLite database, allowing these apps to operate, gather, and save data without the need for an active cellular or internet connection. This was important when choosing a possible technology solution as some potential participants may not have had steady or predictable internet access. These data are stored with no participant information (only a unique participant ID) and can be uploaded whenever a Wi-Fi or cellular connection becomes available. Data transmission occurs using the secure https protocol and is stored in a secure SQL server database accessible only to authorized study personnel via user ID and password authentication. The app itself is protected from unauthorized access by a 4-digit personal identification number (PIN) chosen by the participant. The secure PIN must be entered each time the app is accessed for use, which serves the dual purpose of keeping personal data hidden from others while allowing only the study participant to record data.

#### Phase 2: Planning

Once the technological platform was selected, the behavioral health team began a series of ongoing meetings with the software development team to plan the specifics of the mHealth adaptation. The subject matter experts explained the seven core elements of the in-person WC intervention and worked with the developers to devise the best adaptation methods to retain fidelity to the original delivery mechanisms [22]. The developers then created the first draft of the app intervention screens, which included the *cue card* user interface and embedded video vignettes of success stories from previous WC participants. These drafts were later shown to potential young female participants during the formative research phase to elicit their feedback.

#### Phase 3: Formative Research

Several activities were conducted as part of the formative research phase, including discussions with senior staff from the 3 proposed participating health departments in North Carolina, Community Collaborative Board members, and a series of focus group discussions (FGDs)—2 with young African American women at risk for HIV (N=8) and 6 with service providers who work with young African American women (N=40). Led by the same trained project staff member, all FGDs were audio recorded and had at least one notetaker present. All FGD participants provided written and signed informed consent. Notes and transcripts from each FGD were reviewed for main themes related to the intervention, which included delivery, content, length, and format.

The formative findings indicated that the adapted intervention delivered via mHealth would be well-received among the young women. Young, female focus group participants noted the following:

...certain things like that, that they can relate to that’ll grab their attention.

It’ll help. They’d probably like open up more, be honest...on the tablet versus...talking to somebody.

I feel like just bringing it on a device like that is making it a little bit more up to date. It’s not a video you have to watch. It’s not reading out of a textbook. It’s a little bit more into, you know, today’s time, interactive. I think if you’re really trying to get them to be interested in that, it’s just bringing the attention towards, you know, what they’re interested in today.

Some service providers believed that the mHealth platform might be a way of engaging individuals who are not comfortable discussing certain sensitive topics face-to-face:

...if it’s a meaningful interaction, I think it could play a big role...

They can do it at their own leisure.

...if it’s on...as you mentioned, like a smartphone, then they can do it privately. They can watch it and they can cry if they want to and they don’t have to feel, as mentioned earlier, that they have to be super strong Black women and not express any emotions, even though they feel it.

I think with giving them something concrete to use—on their own schedule, their own time, in their own space will make it effective. It’s not like the normal interventions; this is a little different.

However, service providers also indicated that potential barriers to young women participating and continuing in this study could include lack of motivation to participate or use the mobile app, the amount of information presented to them at once, the length of the sessions, and the potential to use the tablet for other activities being a distraction from completing the intervention. Service providers noted the following about the mHealth platform:

They going be doing everything else except for that app...

They might do one session and dip, but—but at least they did something.

I would prefer it, but I would also be motivated to do it on my own, whereas I don’t know that everyone will do it.

#### Phase 4: App Development

Following the formative phase, the app developers began work on implementing the findings from the planning and formative research phases. Beginning with the aspects of the app that were more unambiguous with respect to adaptation methods, the developers used an iterative and responsive development process to construct the app. This involved rapid communication between the behavioral health and development teams via frequent in-person meetings coupled with more impromptu communication via instant messaging applications such as Microsoft Teams and Skype (Microsoft Inc) for progression and confirmation of app changes and additions to ensure that the content aligned with the source intervention. The team also addressed any early-on *bug fixes* in the app during this phase, such as the correction of programming glitches or content errors.

#### Phase 5: Usability and Pretesting

During this phase, the mHealth development team and various project staff conducted extensive usability testing to ensure the app was intuitive and self-guided. Specifically, members of the behavioral health team meticulously reviewed all the potential pathways of the app using a methodical iterative procedure to test for errors, clarity, and flow. This included going through all options within the app as a potential participant would and documenting any changes needed. Examples of changes requested by the behavioral health team included phrasing of statements, stylistic preferences, and usability enhancements. Continuing the agile development process required that changes and additional bug fixes be implemented by the development team, tested, and redeployed to project staff testing devices.

The pretesting of the mWC intervention was guided by the study staff. A total of 6 study participants who had attended formative FGDs and were eligible for the study were invited to the study team’s main research campus to pretest the near-final mWC intervention on a 7-inch Acer Iconia One 8 project tablet. Participants also completed a satisfaction and usability form for the app and were provided a gift card to thank them for their time.

#### Phase 6: In-house Testing

In the next to last stage of app development, the behavioral health team conducted in-depth testing to prepare for dissemination. Testing of the final intervention was conducted on the target study device: 7-inch Acer Iconia One 8 tablets purchased for the project. The study staff confirmed the basic functionality of all portions of the app, including app behaviors such as adherence to security measures (eg, whether the app required a PIN to access user information), the appropriate progressive disclosure of intervention materials, and acceptable media performance on the tablet.

Throughout testing, the behavioral health team stayed in close contact with the app development team to ask questions, make requests, and identify bugs.

#### Phase 7: Finalization and Maintenance

Once in-house testing was completed, the app development team made a canonical *version 1* build of the mWC app. An initial set of study tablets was provisioned and loaded with the app and then provided to the previously identified study sites in 3 North Carolina health departments.

Over the course of the study, updates to the app were made 5 times. One of the revisions was to implement a method of PIN recovery for participants who had forgotten the required 4-digit PIN they created to access the app. Other revisions were to update and expand the *referral guides* section of the app (refer to the following sections). Whenever the app was updated, the newest version was installed on each new batch of tablets as they were provisioned for the study. In the case of the PIN recovery app modification, the user’s app could be updated on the tablet from a connected computer without the loss of previously collected study data.

### Ethics Approval

This study was approved by the RTI International Office of Research Protection Institutional Review Board (ID Number 13836), in addition to the research committees of Wake County Human Services and the Durham County Department of Public Health. The Guilford County Department of Public Health Director granted approval in lieu of a formal review by a research committee.

## Results

### Overview

When setting out to design the app-based version of the WC, it was important to the researchers that the mobile adaptation closely matched the original version of the WC to enable a faithful comparison between the effectiveness of the two modalities. As previously stated, the in-person intervention sessions comprise health information content delivered via cue cards followed by a behavioral goal-setting process, both guided by a trained project staff member. We decided to keep the content and delivery modes of the sessions the same where possible and to modify only as necessary for presentation on a mobile device. Although the decision to provide participants with study-issued devices was made early on, tablets were chosen over smaller devices, such as smartphones, to provide a better user experience for the rich media of the intervention.

The primary difference between the two delivery methods is the self-directed aspect. The in-person intervention is guided by a trained interventionist, whereas the mobile version is almost entirely self-paced and self-guided. In the app, participants choose options from menus and can move forward and backward as they please, with the opportunity to replay any portion of the app at any time. Although repeating the consumption of content and time spent in sessions is largely up to the participant while using the mobile app, the linear nature of the intervention sessions is preserved by providing access to subsequent sessions and related activities only as the previous ones are completed. Table 1 presents a comparison of the two delivery methods regarding components and characteristics.

**Table 1:** Comparison between the two delivery methods of the WC^a^.

Components	Face-to-face WC	mWC^b^
Timing	This includes 2 sessions, typically scheduled a week apart.	Session 2 is immediately available to the participant upon completion of session 1.
Interventionist or navigation of intervention	Sessions are one-on-one between a trained project staff member and a study participant.	Sessions are self-guided by using forward and backward navigation to move between slides, videos, and activities. Each card is accompanied by an audio narration reading the content.
Order of content	Cue cards and video vignettes are reviewed in a specified order by the interventionist.	Cue cards and videos are presented in a specified order via programming.
Activities and participant engagement	Key ideas are discussed as they are presented, with back-and-forth communication to aid in knowledge retention.	Key ideas have been developed into accompanying *activities* that intersperse the cue cards. These are interactive *minigames* the participant completes to aid in knowledge retention.
Role-play and rehearsal	Scenarios such as condom negotiation and application are practiced during role-play to allow the participant to develop skills.	Cue cards prompt participants to consider how they might react in certain situations. There are also *follow along at home* videos presented, during which participants are encouraged to practice along.
Personalized action plan	At the end of each session, the participant develops their action plan with guidance from the interventionist.	At the end of the session, the participant constructs their action plan by reviewing a series of screens with common goals and steps from prior studies.
Revisiting action plan or check-in	In session 2, the participant and interventionist discuss progress on the participant’s action plan from session 1.	Once an action plan is confirmed by the participant, a new menu item becomes available on the home screen. Participants are encouraged to visit this *monitor goals* screen often to reflect on and update their action plan.
Check-in	After session 2, the interventionist follows up with the participant to check on the progress of their action plan.	After session 2, the participant unlocks an additional set of potential goals to add to their action plan. The *monitor goals* screen tracks all action plan goals in one place and can be visited at any time.

^a^WC: Women’s CoOp.

^b^mWC: mobile Women’s CoOp.

### Cue Cards and Video Media

**Figure 1:**
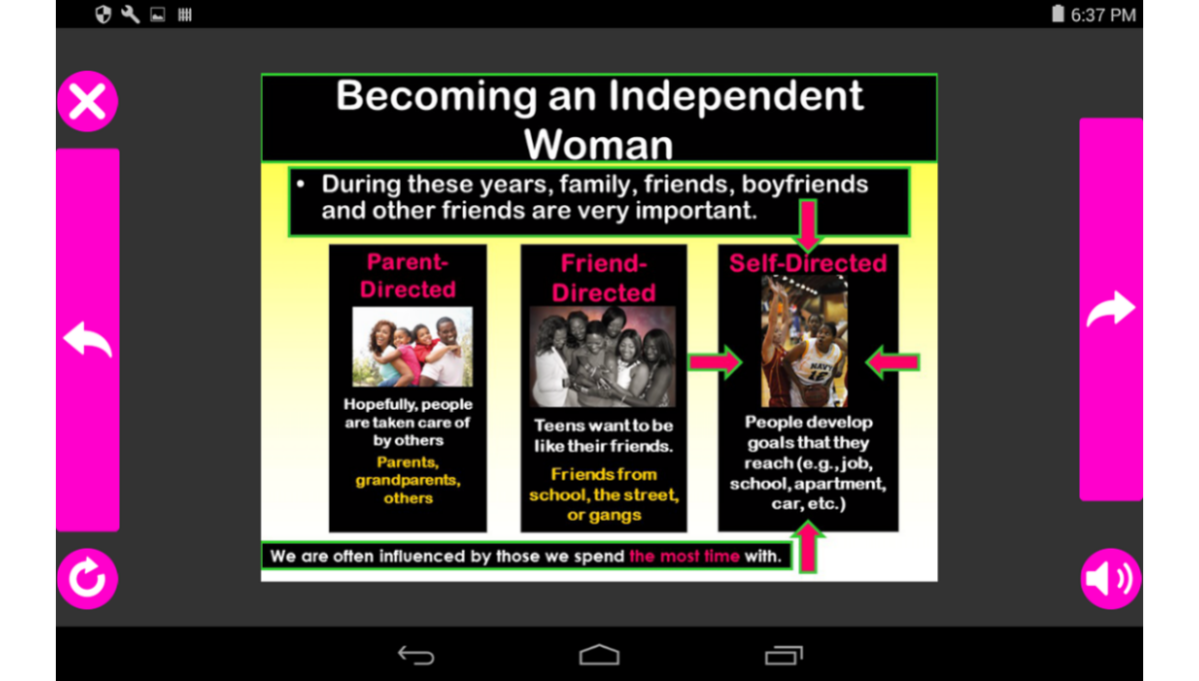
Sample cue card.

The bulk of the health information content was adapted to a self-directed slideshow by converting the Microsoft PowerPoint slide decks, or cue cards, originally presented in person by an interventionist. The participant enters a session by choosing it from the home screen menu and is placed into an engaging full-screen presentation (Figure 1).

Cue card design, content, and order are the same in the face-to-face WC and the mWC interventions. However, in the mWC app, the participant moves through the cards at their own pace by tapping forward and backward buttons. The participant could also choose to pause or exit the app at any time and pick up later where they left off. Each card’s content is narrated by embedded audio narration and can be replayed if desired. The entire session itself is available for play or replay from the home screen menu at any time after the participant has unlocked it by completing the previous session or goal-setting activity (action plan).

**Figure 2:**
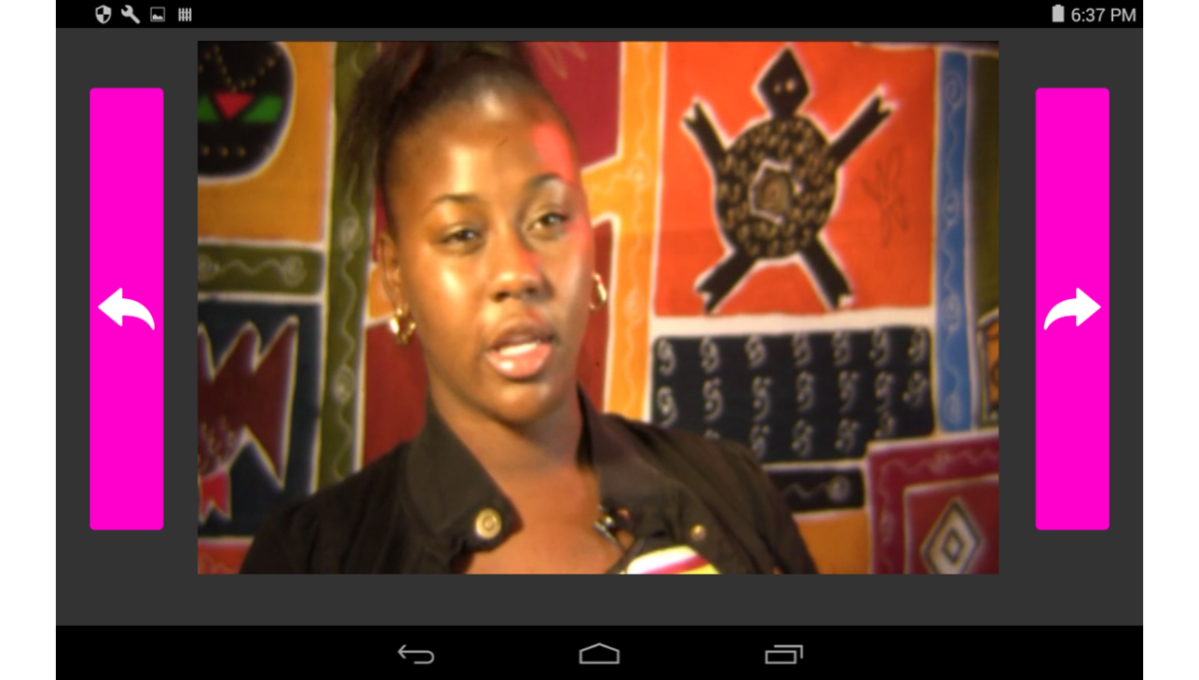
Sample frame from a video vignette.

Video vignettes of mostly African American young women sharing brief personal stories relating to the session topics are interspersed throughout the text and image-based cue card content (Figure 2).

Owing to concerns about future scale up and dissemination, the amount and file size of videos were reduced. At the time of development, the Apple App Store limited the size of apps to 100 MB if they were to be downloadable over a cellular connection.

### Referral Guides

Participants in the in-person intervention received referrals, as needed, to local organizations or resources providing services in the domains of food aid; employment, education, housing, legal, parenting, and substance use; sexual, mental, and general health; and domestic violence and sexual assault support.

**Figure 3:**
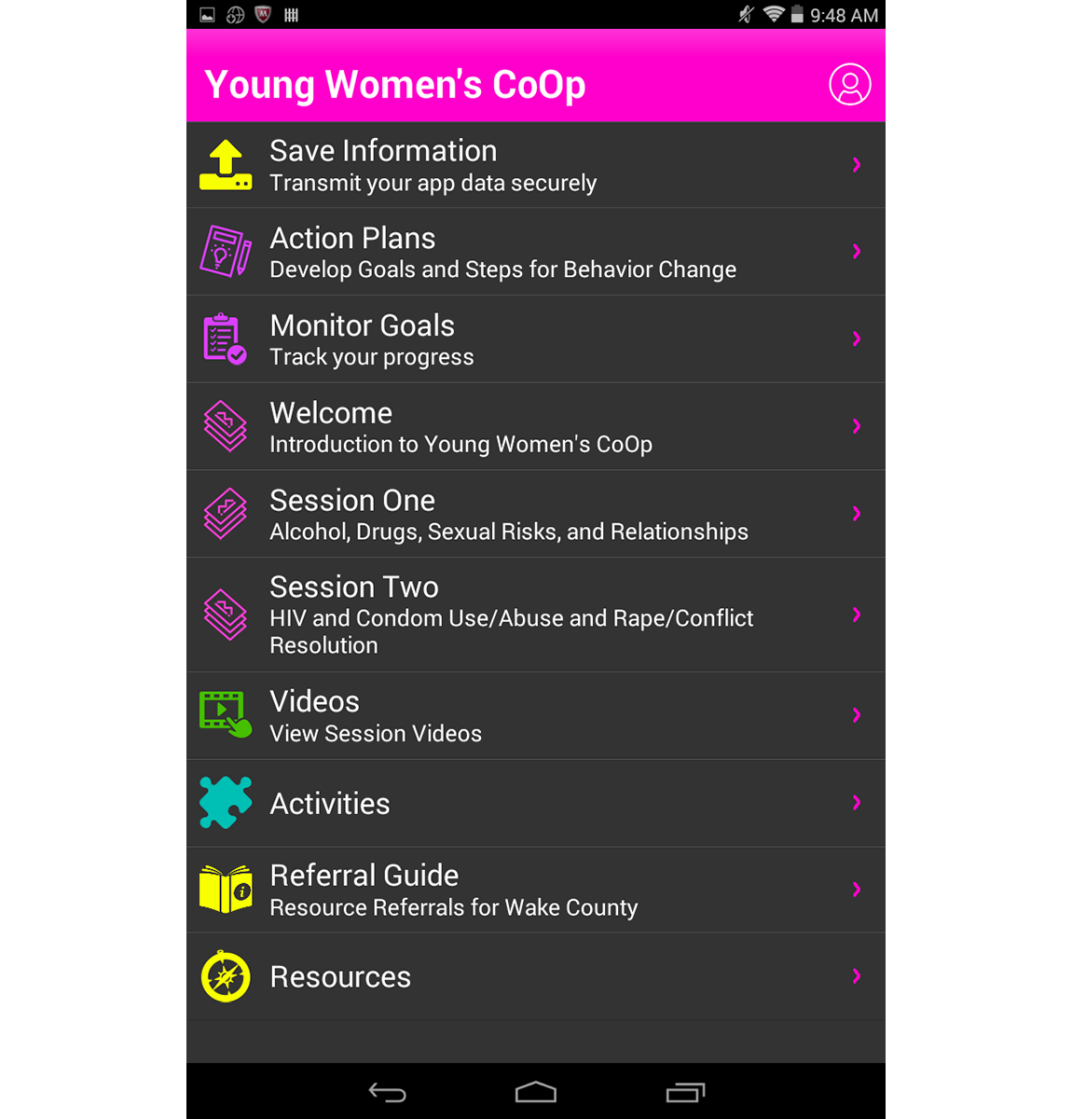
Home screen with the referral guide available.

The same resources are provided in the app but with a self-directed aspect. A *referral guide* option is always available from the home screen menu (Figure 3) and participants choose which geographical location (out of the ones provided based on the location of the study health clinics) is most convenient to them.

As referral resources are updated by health staff, the app is also updated with new information. Participants joining the study would receive the most up to date version of the app during their scheduled mHealth appointments.

### Adaptation to the mHealth Format: Interactivity

#### Interactive Activities

Although adapting a more passive activity such as consuming information by following along with cue cards or watching videos is relatively straightforward, replacing the back-and-forth communication during knowledge retention activities in the in-person intervention provided a greater challenge. The goal of this aspect of the behavioral intervention is to recap previously presented key topics in a reciprocal manner with the aim of assessing understanding while reinforcing recall and absorption of crucial information. Therefore, 3 *minigames* and 2 *follow-along* video activities were developed to take the place of the person-to-person interaction present in the original WC and adapted WC interventions. Similar to the video vignettes, these activities are interspersed throughout the cue card presentations.

**Figure 4:**
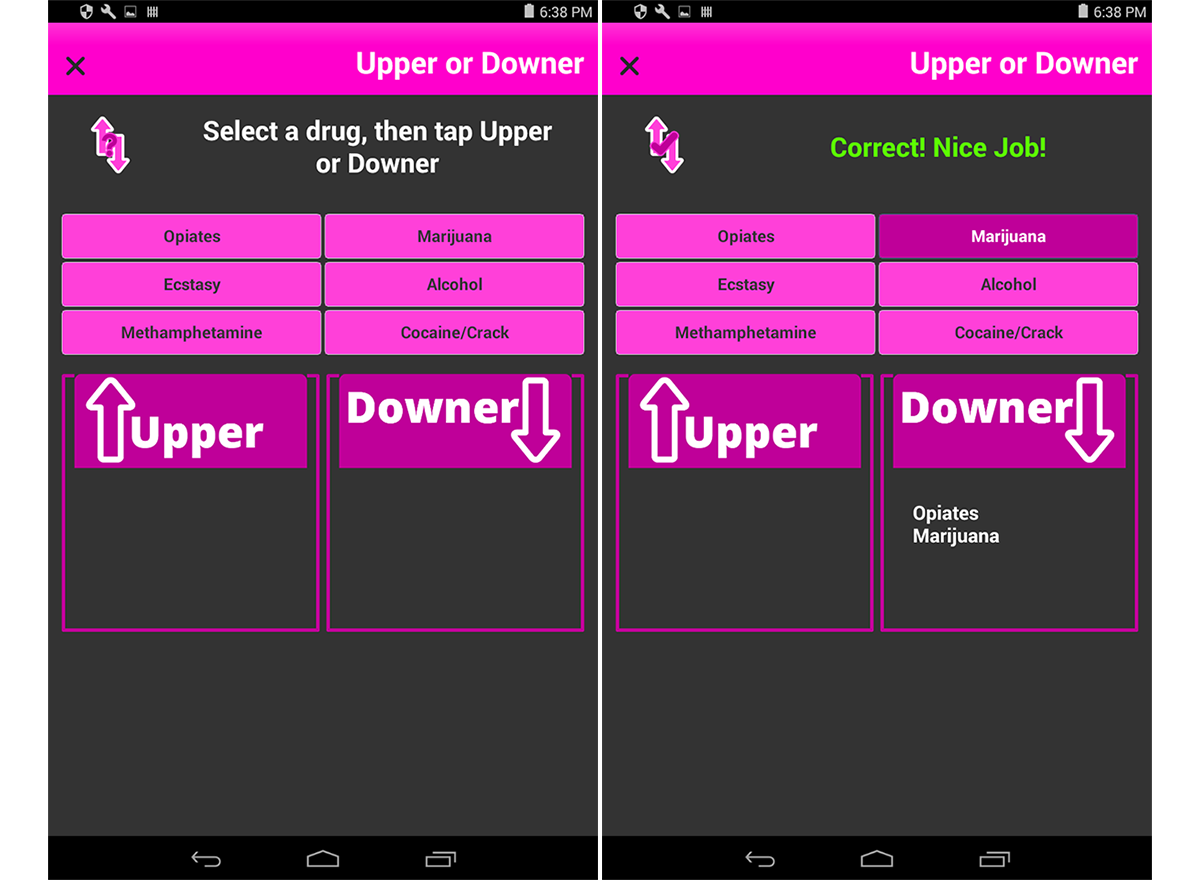
Two screens of the Upper or Downer activity.

The minigames present the participant with an exercise to complete and provide real-time feedback about how well they are doing. The *Upper or Downer* activity (Figure 4) reviews topics about alcohol or other drugs and tasks the participant with sorting a list of previously discussed illicit drugs into their respective category of *upper* or *downer*. Each correct answer is rewarded with a checkmark and a notice of *Correct! Nice Job!*; incorrect answers elicit a message for the participant to try again. Once all drugs have been sorted, the participant sees their final score.

Similarly, the *Levels of Risk* minigame recaps sexual risk information and asks participants to sort a mixed-up list of sex acts into order from *least risky* to *most risky*. The third minigame, *Myths and Truths*, assesses participants’ recall of the veracity of some commonly held but erroneous beliefs about violence against women by posing the question “Myth or Truth?” In addition to the minigames, there are 2 follow-along videos that demonstrate male and female condom mastery. The videos show the process of removing a male or female condom from its wrapper, its application (on a banana or hand stand-in, respectively), and its proper removal and disposal. Participants are encouraged to practice along with the video using condoms provided by the study when they receive the tablet [24].

#### Action Plans

**Figure 5:**
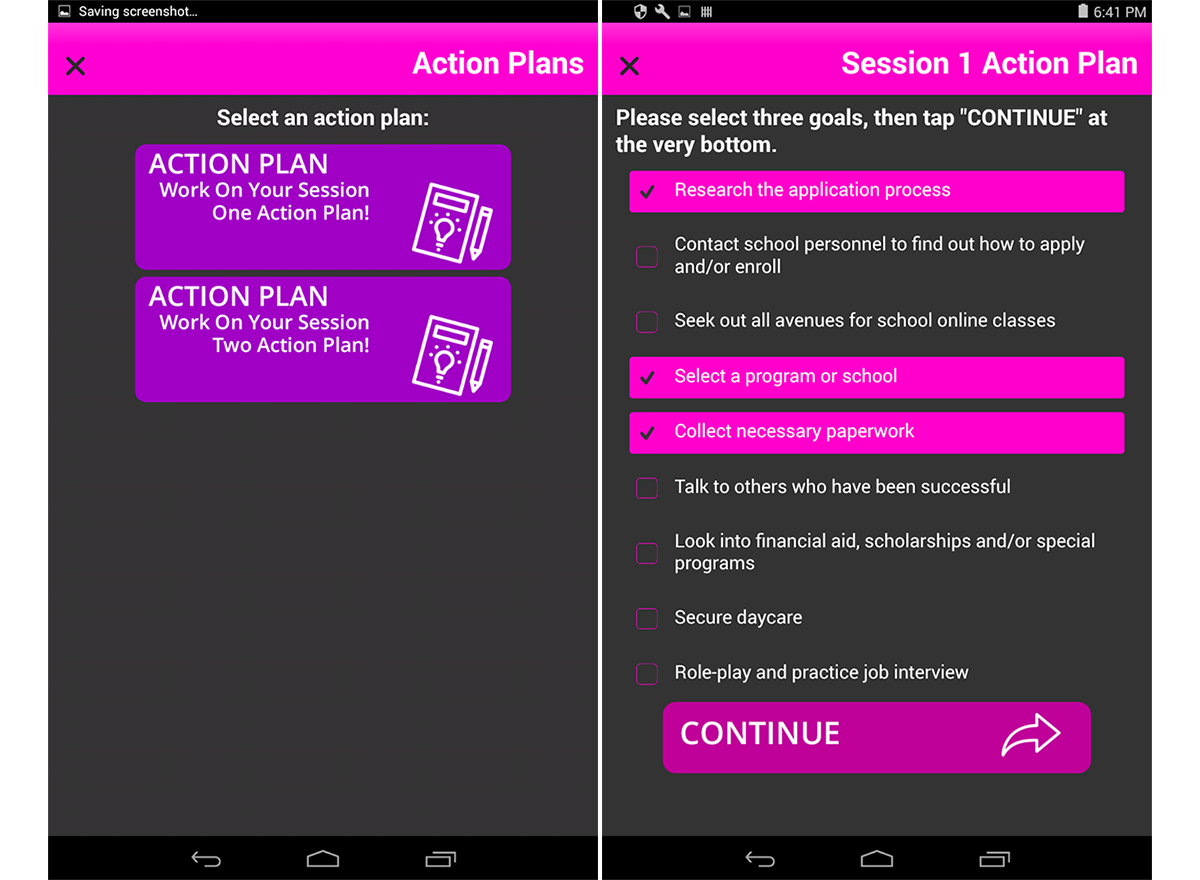
Two screens from the Action Plans activity.

Another important component for the mWC adaptation was translating the personalized risk-reduction action plan for the mHealth app. At the end of each session, participants in the in-person WC work with the guidance of the interventionist to set goals related to the intervention material and create specific and concrete steps to reach these goals. In the absence of the advice and direction of trained health staff, the app provides the participant with a list of session-related objectives curated from a review of a random sample of common goals and steps recorded on hard-copy action plans from a previous North Carolina WC intervention study with adolescents (Figure 5) [28].

Participants are instructed to review the goals presented to them and choose 3 goals to work toward from each topic domain (eg, in session 1, goals are divided into two categories: alcohol and drug use and life improvement). Once they have selected their goals, they review them on a confirmation screen and tap a button to confirm their goals. Once their goals are confirmed for the session’s categories, a new menu option appears on the home screen—*Monitor Goals*. Participants are encouraged to come back frequently to review or revise their goals or choose new goals when the previous goals have been met.

#### Reminder Notifications

As part of the face-to-face WC, participants are called in advance of their next session to remind them to attend. Similarly, for the mobile app, periodic reminders were programmed to pop up in the form of notifications on the tablet; the participant is able to set the frequency of these notifications during their mHealth introduction appointment. As these notices could potentially be seen by anyone looking at the tablet without the need to log into the app, they were developed to be generic to protect the privacy and confidentiality of the participant. An example notification was, “Don’t forget to visit the app!”

## Discussion

### Principal Findings

The disparity in HIV and other STI rates among young African American women in the US South, when compared with other groups, continues to be a public health concern. Targeted behavioral health interventions such as the WC have been shown to be helpful but are costly to conduct in person, and the time and resources required to participate can sometimes be prohibitive for those in the intended populations.

Owing to the COVID-19 pandemic, health resources are even more strained, which makes a new application more feasible and acceptable. The increased adoption of mobile devices has opened a potential avenue for reaching a greater number of young women at risk for HIV and other STIs. Public health practitioners have the opportunity to adapt evidence-based interventions to an mHealth format and possibly reach more individuals than ever at a lower cost. However, it is still largely unknown how these adaptations are received by the intended populations. Will the decreased in-person interaction be detrimental? Or perhaps the innovative and efficient media format of an mHealth app will be of more interest to younger age groups.

### Limitations

For future studies, it may not always be possible to provide participants with an electronic device (eg, tablet) to complete the intervention. However, in this study, it was not a confounding factor, as participants were not excluded because of a lack of access to a tablet.

### Conclusions

In the case of the WC to mWC adaptation process, the development team wanted to mirror the in-person intervention as closely as possible for fidelity and provide a true comparison of the two formats. Future research could adapt this approach further, such as by potentially using more interactive gamification components, including rewards and points for completing intervention activities, to encourage engagement and retention in intervention content. This first adaptation of the mWC app showed promise for participant engagement with a mobile app modality and may pave the way for future participant recruitment and retention strategies, more accessible knowledge dissemination, and practicable participant behavior change via mobile gamified behavioral intervention activities.

## Abbreviations

FGD: focus group discussion
mHealth: mobile health
mWC: mobile Women’s CoOp
PHIT: Personal Health Informatics and Intervention Toolkit
PIN: personal identification number
STI: sexually transmitted infection
WC: Women’s CoOp
